# Noise-induced hearing loss in farmworkers: a scoping review

**DOI:** 10.3389/fpubh.2025.1502489

**Published:** 2025-03-03

**Authors:** Laura Coco, Marisa Fried, Obdulia Loria, Lluvia Vazquez, Katherine Ekonomo, Gabriela Sanchez, Annie J. Keeney, Cheryl L. Beseler

**Affiliations:** ^1^School of Speech, Language, and Hearing Sciences, College of Health and Human Services, San Diego State University, San Diego, CA, United States; ^2^School of Social Work, College of Health and Human Services, San Diego State University, San Diego, CA, United States; ^3^Department of Environmental, Agricultural, and Occupational Health, College of Public Health, University of Nebraska Medical Center, Omaha, NE, United States

**Keywords:** noise-induced hearing loss, farmworkers, occupational health, hearing conservation, scoping review

## Abstract

**Objective:**

Farmworkers who have prolonged exposure to loud noise are at risk for disabling hearing loss. The objectives of this scoping review are to (1) identify and summarize the evidence on noise-induced hearing loss in farmworkers, (2) describe instruments used to evaluate hearing loss outcomes, (3) describe testing approaches and limitations, and (4) provide recommendations for future studies that seek to quantify hearing loss in this population.

**Methods:**

We performed a systematic search of three electronic databases, PubMed, CINAHL, and Scopus, to identify articles related to noise-related hearing loss in farmworkers. Our search was guided by Arksey and O’Malley’s methodological framework and PRISMA-ScR guidelines.

**Results:**

A total of 57 articles met inclusion criteria. The majority of studies were undertaken in North America (*n* = 32, 56%), and most were in the midwestern United States. Farmworkers tended to be white, male, and work in crop agriculture. A total of 47 studies (82%) used audiometry to measure farmworkers’ hearing sensitivity, among which testing was carried out at various locations, including hospitals, clinics, farmworkers’ homes, and worksites. The criteria for defining hearing loss varied across studies making it difficult to summarize results. Among 14 studies that used a cutoff point greater than 25 decibels, the prevalence of hearing loss ranged between 46 and 98%. Subjective outcomes (used in 14 studies) were typically assessed using a variety of researcher-developed questions. The prevalence of hearing difficulties in this category was as high as 87%.

**Conclusion:**

Hearing loss is prevalent across studies and does not appear to decrease over the years. Our findings call for more research among diverse farmworker populations. Further, given the high prevalence of hearing loss in many of the studies reviewed, there is clearly a need to develop strategies to protect farmworkers from noise exposure and noise-induced hearing loss.

## Introduction

Agriculture is among the most hazardous industries (1). Farmworkers are exposed to numerous occupational health and safety hazards, including physical and biomechanical injuries, mental and emotional problems, chronic diseases, including cancer due to exposures at work, and even death. Although less discussed, agricultural work can also involve excessive levels of noise, putting farmworkers at significant risk for permanent and disabling hearing loss (2–4). Farming activities that present high levels of noise include the operation of machinery, such as power tools, tractors without cabs, and older cabbed tractors, as well as the handling of livestock (5, 6).

Repeated and prolonged exposure to noise leads to noise-induced hearing loss (NIHL), a permanent and irreversible condition. NIHL typically impacts the ability to hear high-frequency sounds, severely impacting speech understanding and communication and thus negatively affects health-related quality of life. Farmworkers who are co-exposed to both noise and ototoxic chemicals such as herbicides and pesticides (7) may be at risk for more severe NIHL through a synergistic effect (8–10). In addition to the risk of NIHL, elevated noise levels can increase the risk of work-related injuries and worker stress (11–13).

Occupational noise standards differ across the world, and many countries have not adopted legislation regarding permissible noise levels (14). In the US, the Occupational Health and Safety Organization (OSHA) requires employers to implement a hearing conservation program when workplace noise meets or exceeds 85 decibels on the A scale averaged over a period of 8 h (15). Hearing conservation programs include measures such as serial monitoring of noise and hearing loss, limiting noise exposure, and using personal hearing protection. However, the hearing conservation clause does not apply to all the agricultural workforce, and the degree to which noise control is enforced in this sector is unknown.

There are no global or national (US) estimates about farmworkers’ hearing ability, although the US prevalence of hearing loss in agriculture, forestry, and fishing industries combined is 16% (16). Regional studies focused specifically on farmworkers report even higher rates of hearing loss, ranging from 36 to 47% (5, 17–23). By comparison, two other high-noise industries—construction and manufacturing—have estimated hearing loss prevalence rates of 23 and 20%, respectively, while quieter occupations, such as couriers and messengers, have a lower prevalence of 8% (16).

According to the most recent U.S. Census of Agriculture (2022), there are 1.9 million farms across the country, the majority of which are small operations of less than 49 acres, though some farms exceed 5,000 acres. The average farm size in the U.S. is 463 acres. Texas leads the nation with more than twice as many farms as any other state (24). Insights from the National Agricultural Workers Survey (NAWS) (2021–2022), which included interviews with 2,598 crop workers, reveal a predominantly male workforce (68%) with an average age of 39 years, the vast majority of whom (98%) are Spanish speakers (25).

Research has focused on the health and safety of farmworkers for decades due to the physically demanding and often hazardous nature of agricultural work. Farmworkers face various and disproportionate health risks, including exposure to pesticides, heat stress, musculoskeletal injuries, and respiratory issues. Several recent literature reviews have explored farmworkers’ acute and chronic health issues, including kidney disease (26, 27), musculoskeletal injuries (28), substance use disorders (29), as well as psychosocial wellbeing (30, 31). Hearing problems have historically received less attention, although a recent review examined the relationship between hand/arm vibrations and hearing loss among agricultural and forestry workers (32). The only known review to examine the prevalence of farmworkers’ hearing abilities, which included 15 studies published between 1958 to 2001, was conducted by McCullagh (33). This paper updates McCullagh’s review, examines progress over the past two decades, and offers future research and practice recommendations. Additionally, we expand the review’s scope to include farmworker populations beyond the US.

## Methods

### Overview

This scoping review investigated research on hearing loss among farmworkers, summarizing prevalence estimates, methodologies, and barriers to hearing testing in this population. Among the many possible review types, a scoping review was most appropriate for this research question (34). A scoping review is a tool for determining the volume of literature on a particular subject and allows researchers to identify knowledge gaps, clarify concepts, and is helpful in understanding whether more specific questions can be posed (35).

To ensure transparency and reproducibility, we developed a protocol *a priori* and followed the PRISMA-ScR (Preferred Reporting Items for Systematic Reviews and Meta-Analyses extension for Scoping Reviews) checklist (Supplementary Appendix Table 1) to conduct the review (36). The methodology for this review was guided by the framework described in (37) and further refined by Levac et al. (38). This methodological framework broadly includes developing a research question, extracting information from relevant studies, and descriptively and interpretively analyzing the data. Specifically, the stages include (1) identifying the research question, (2) formulating a search strategy plan and identifying relevant studies, (3) study selection based on inclusion and exclusion criteria, (4) charting the data according to variables of interest, and (5) collating, summarizing, and reporting the results numerically as well as qualitatively using a thematic analysis approach. The team participated in weekly in-person check-in meetings and interim discussions via email and chat-based messaging platforms.

### STAGE 1: Identifying the research question

The research question was developed using the PCC mnemonic (Population, Concept, Context) as a guide (39). Table 1 illustrates the PCC outline that frames our research question: What is the extent of hearing loss in farmworkers, and what testing methods are used? As there is no known commonly accepted definition for farmworker (40), our search strategy was purposefully broad.

**Table 1:** Population, concept, context framework for our scoping review on farmworkers’ hearing loss.

Inclusion criteria	Exclusion criteria
**Population:** Farmworkers of any type including nursery workers, dairy farmers, livestock, and crop workers Any age Any sex/gender Any race/ethnicity Including migrant and seasonal farmworkers	Fisheries Exclusive focus on family members or community members who are not farmers
**Concept:** The article either involved primary data collection (i.e., the authors collected the data themselves in order to answer their research question) or secondary data analysis (i.e., the authors did not collect the data themselves but used an existing dataset or census data). The outcome(s) investigated were hearing loss related outcome(s). This could include self-report survey, objective (audiometric), or qualitative outcomes.	Systematic reviews Meta-analyses Not peer-reviewed
**Context:** All settings and geographic locations are included	Full text not available in English

### STAGE 2: Identify relevant studies

#### Search methods

Author LC and a health sciences research librarian designed the literature search strategy. The goal of the search was to understand the scope of the literature related to the prevalence of hearing loss in farmworkers worldwide, and thus, the search strategy was purposefully broad. Table 2 provides a detailed overview of the search. A primary search was performed in October 2023, and an updated search was conducted in June 2024 to enhance the timeliness of the results.

**Table 2:** Database search strategy.

Databases	Search strategy
PubMed	(“Hearing Loss, Noise-Induced” [Mesh] OR “noise-induced hearing loss” OR “noise induced hearing loss”) AND (Farmers [Mesh] OR farmers OR “farm workers” OR farmworkers OR agriculture)
CINAHL	(“Hearing Loss, Noise-Induced” [Mesh] OR “noise-induced hearing loss” OR “noise induced hearing loss”) AND (Farmers [Mesh] OR farmers OR “farm workers” OR farmworkers OR agriculture)
Scopus	(“hearing loss, noise induced” OR “noise-induced hearing loss” OR “noise induced hearing loss”) AND (farmers OR “farm workers” OR farmworkers OR agriculture)

#### Data sources

Three databases were searched: PubMed, CINAHL, and Scopus. Three researchers also hand-searched reference lists of included articles to identify any additional relevant studies not captured by the database search. Citations identified by the search terms were imported to Zotero Citation Manager 6.0, and duplicates were identified and removed. The references were then imported into Rayyan, an online software for literature reviews (41).

### STAGE 3: Study selection based on inclusion and exclusion criteria

Articles were included if they provided a count or prevalence proportion estimate of farmworkers’ hearing loss or if they included data on subjective hearing difficulties. Qualitative studies were also included if they addressed farmworkers’ hearing loss. No time limit for the year of publication was imposed. Articles must be available in English. We included secondary data, and case studies. Commentaries were not included. Both children and adults were included in the study, with no exclusions based on race or ethnicity.

#### Screening and study selection

We used a two-phase approach for study selection: (1) titles and abstract screening followed by (2) full-text review of identified articles. For the first phase, three authors (OL, MF, and GS) independently screened all titles and abstracts according to inclusion and exclusion criteria. After title and abstract screening, relevant articles were procured for full-text review. Four researchers (OL, MF, GS, and KE) reviewed full texts against the inclusion and exclusion criteria. The full-text review also included relevant papers that were added from the hand-search and any titles that lacked abstracts. For both phases, consensus was reached through discussion with the primary author (LC).

### STAGE 4: Charting the data according to variables of interest

Data were charted using a spreadsheet that focused on the scoping review question. We also included information on methods, study location, year, and methods for evaluating hearing loss. As per the recommendations in Levac et al. (38), two reviewers piloted the data charting sheet, each extracting data from 10 included full-text records (20 total). During weekly meetings, project team members refined the data charting form. The remaining articles were then reviewed, and data was extracted by four researchers (OL, MF, GS, and KE) who each reviewed an equal number of articles. Author LC checked validity and completeness.

## Results

### STAGE 5: Collating, summarizing, and reporting the results

Detailed charting is provided in Supplementary Appendix Tables 2–4 and an overview of the results are summarized in Table 3.

**Table 3:** Overview of key findings from 57 studies on noise exposure and hearing outcomes in farmworkers.

**Key Fi‌ndings**	**Details and examples**
**Hearing Loss by Age**	Hearing loss increased with age, e.g., 25% of males had hearing loss by age 30, 50% by age 50; hearing thresholds worsened by ~4dB per decade of age.
**Tractor Drivers**	Worse hearing at 1kHz, greater "dips" at 4kHz; high-frequency hearing loss (e.g., 50% vs 10% in control group); hearing loss >25dB associated with driving >15 years.
**Other Professions**	Hearing loss generally higher in farmworkers compared to other professions (e.g., office workers).
**Noise Sources**	Significant noise sources: tractors, pig breeding, pesticides, hunting, ATVs, and chain saw use.
**Gender Differences**	Men had more hearing loss than women, particularly at higher frequencies and with age.
**High-Frequency**	High-frequency PTA was more prevalent (e.g., 78% vs 17% control group); worsened hearing loss at 3-6kHz.
**Self-Reported Hearing**	Mixed agreement with audiometric data; self-reported difficulties ~35%-87%.
**Left vs Right Ear**	Left ear showed more severe loss than right ear in multiple studies.
**Farm Youth**	Farm youth had a higher prevalence of hearing loss compared to controls.
**Pesticide Exposure**	Associated with increased risk of hearing loss.
**Work Duration**	Hearing loss strongly correlated with years worked in agriculture.
**Interventions**	Hearing loss improved after educational interventions in certain studies.

### Descriptive characteristics of included studies

**Figure 1:**
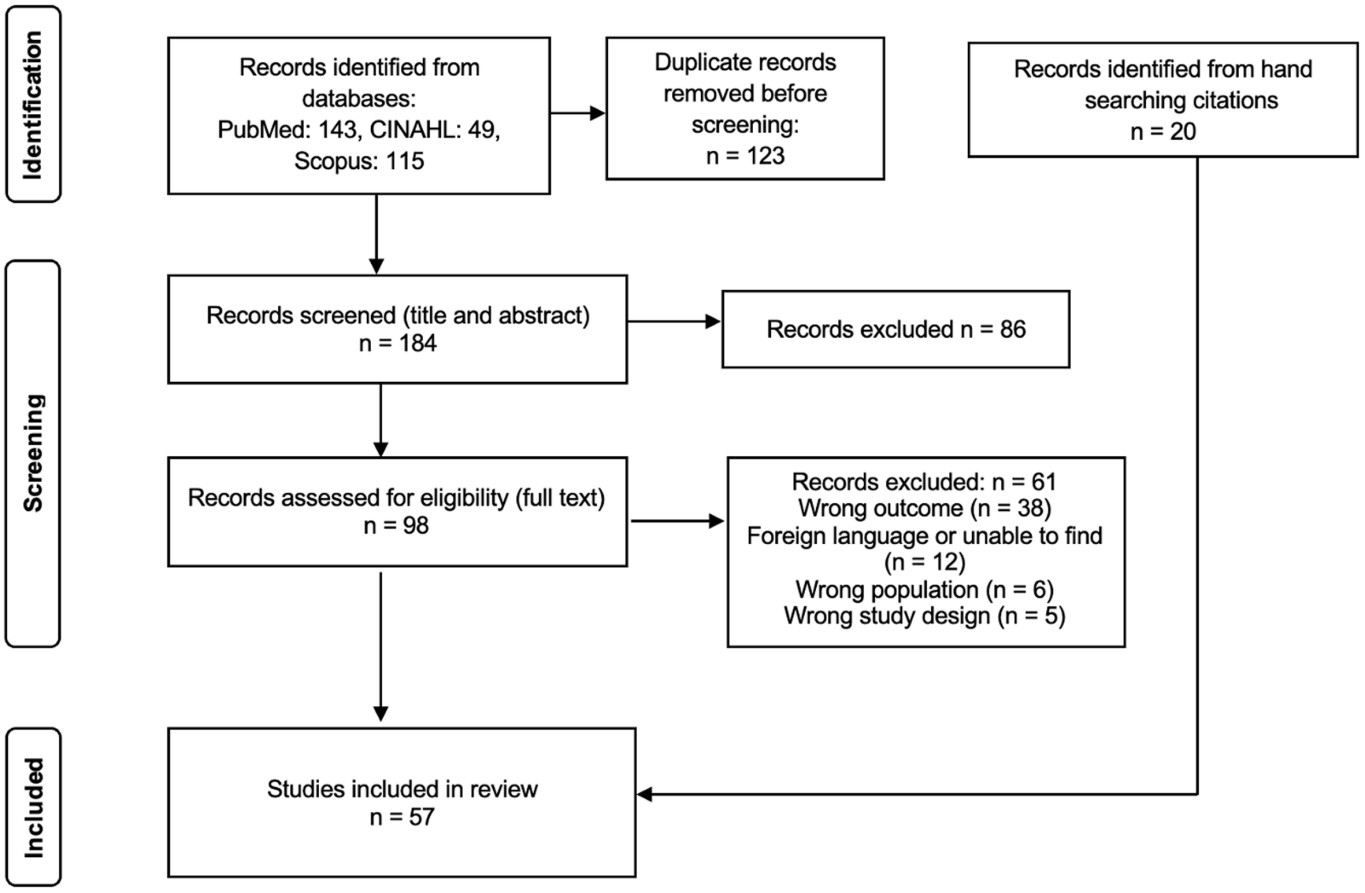
Flowchart of selection process for included studies.

**Figure 2:**
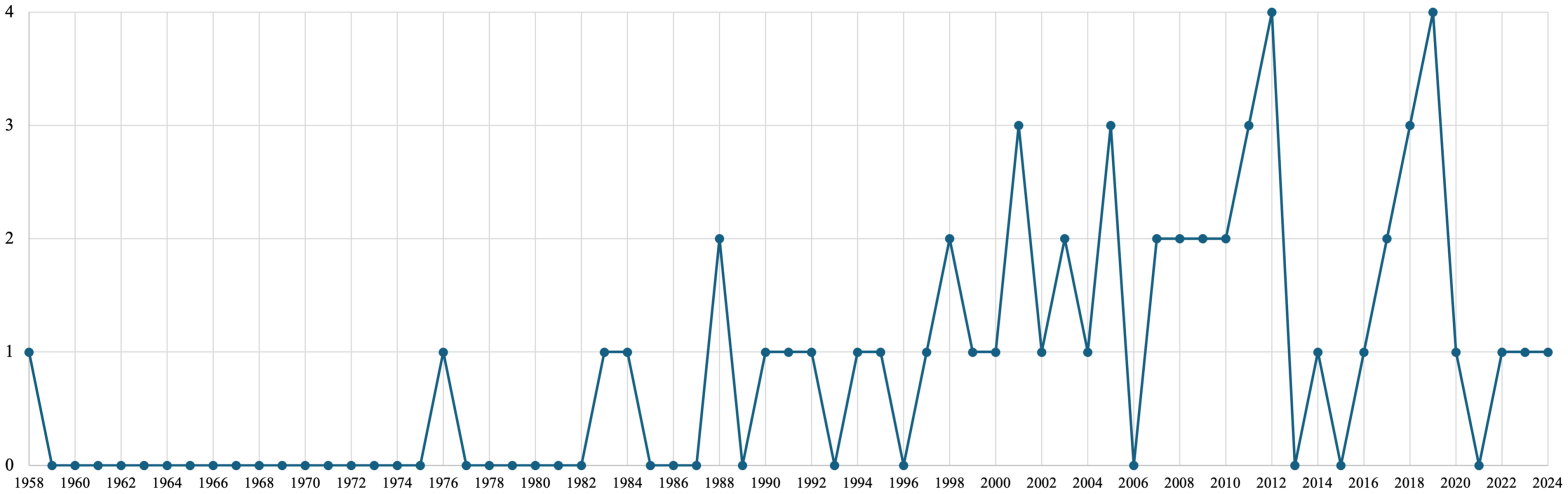
Number of publications on noise-related hearing loss in farmworkers by year.

The article inclusion flowchart is illustrated in Figure 1. A total of 57 published articles met the inclusion criteria and were included in the final review. As shown in Figure 2, the study period spanned from 1958 to 2024, during which the number of publications per year on hearing loss in farmworkers ranged from zero to four. Results reveal a slight increase in publications after 2001, although there have been relatively few publications on farmworkers and hearing loss in the last few years. The most publications were in 2012 and 2019, with four articles published each year.

### Study designs

The majority of studies were cross-sectional, one-group prevalence studies. Three studies used a longitudinal design to evaluate the effect of an intervention on hearing outcomes. Two of these studies found no change in hearing outcomes following the intervention (42, 43), and one study found that male farmworkers had more significant asymmetry and more significant hearing loss when compared to female farmworkers after a 16-year follow-up interval (44). Other studies used a longitudinal design to evaluate hearing changes over time and did not involve an intervention. For example, Renick et al. (45) evaluated farm adolescents’ audiometric thresholds at two intervals, approximately 10 years apart. Less than half of the studies (*n* = 24, 42%) compared farmworkers’ hearing abilities with other groups, typically those not exposed to agricultural noise, such as a population of age-matched office workers (46). Two studies used a case report design (47, 48). The remaining study’s design was not described (49).

### Populations

The median sample size across all studies was 156, ranging from 1 to 360,000. Excluding large-scale public health surveillance studies, the median sample size for the remaining studies was 90. The average age of farmworkers across studies was 46.5 years (SD = 7.5). Seven studies included adults and adolescents; four focused on only adolescents, with the remainder including adult farmworkers. The average proportion of males in the included studies was 80% (28 to 100%). Only two studies reported more female farmworker participants compared to males (50, 51). The majority of studies did not report farmworkers’ race/ethnicity. Among the studies that did report this variable (*n* = 10, 18%), eight reported that the farmworkers were ≥ 97% White or European. Two studies included primarily Hispanic/Latino farmworkers (18, 52).

**Figure 3:**
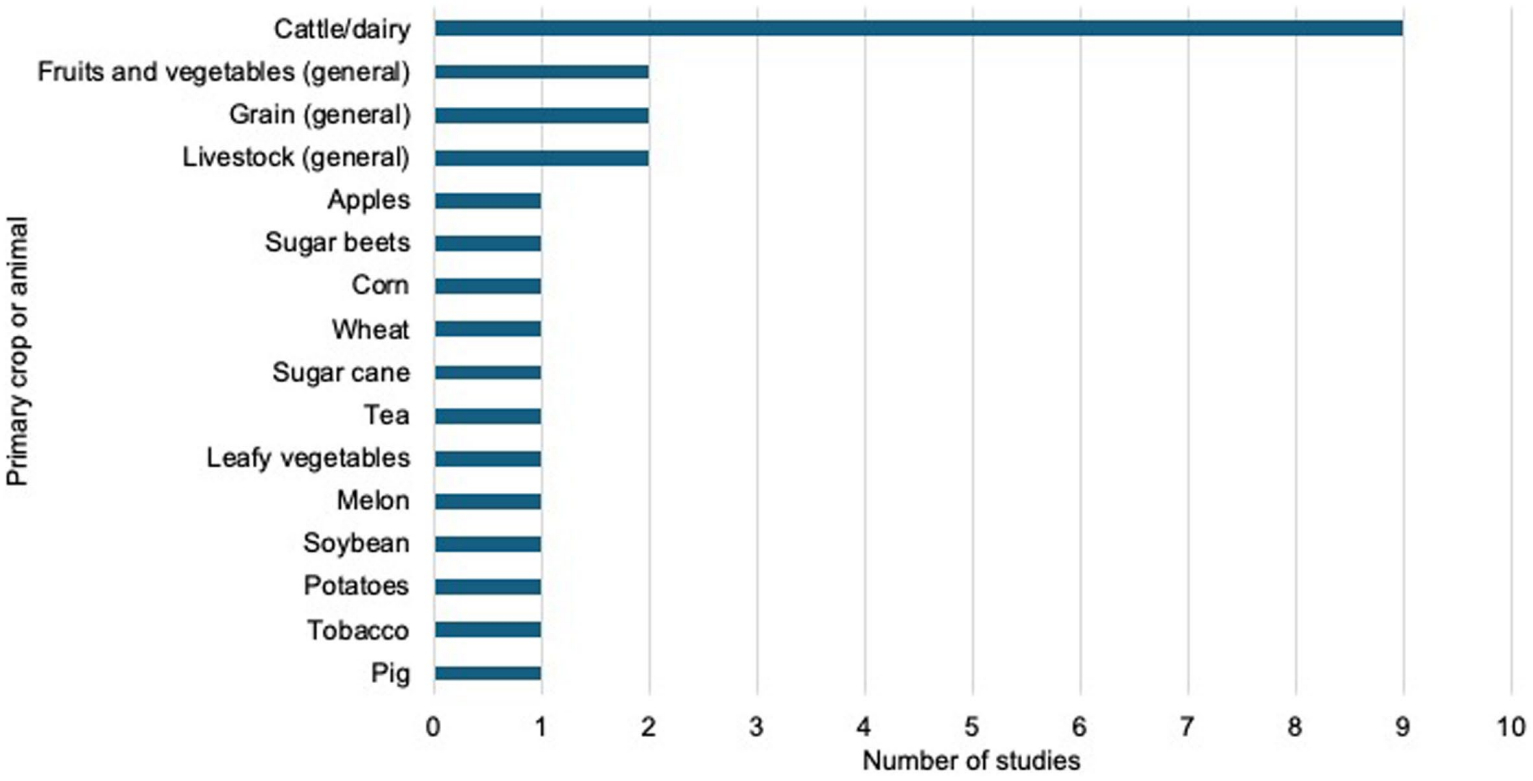
Number of publications by primary crop or animal.

Most articles did not provide information on the primary area of agriculture that farmworkers worked in. Among those that did provide this information, most studies focused on individuals who worked in crop agriculture (*n* = 10, 18%), including potatoes, grain, tobacco, sugar cane, melon, and leafy vegetables, among other kinds of crops. Fewer studies focused on individuals who worked in livestock agriculture (*n* = 4, 7%). Nine studies included farmworkers in both crop and livestock agriculture (*n* = 9, 16%). Two studies focused on farmworkers who drive tractors and did not indicate their area of agriculture. Figure 3 illustrates the number of studies by area of primary crop or animal.

**Figure 4:**
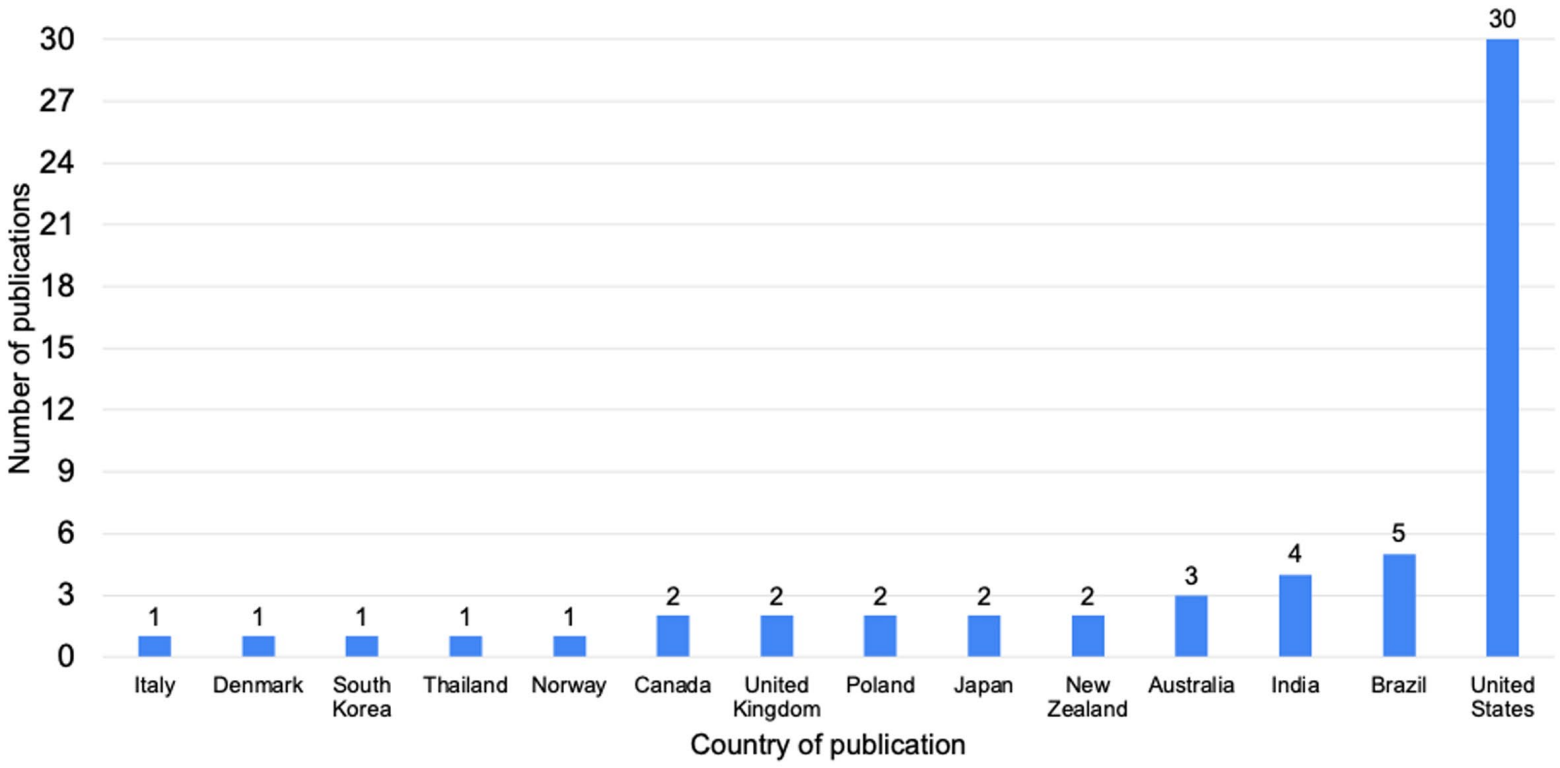
Number of publications by country.

The majority of studies originated in the US (*n* = 30, 53%). Studies outside of the US included the countries of Brazil (*n* = 5, 9%), India (*n* = 4, 7%), Australia (*n* = 3, 5%), New Zealand (*n* = 2, 4%), Japan (*n* = 2, 4%), Poland (*n* = 2, 4%), the UK (*n* = 2, 4%) and Canada (*n* = 2, 4%). One study was identified from the following countries: Norway, Thailand, South Korea, Denmark, Canada, and Italy. Within the US, eleven states were identified: Ohio, Michigan, Missouri, Wisconsin, New York, Iowa, Louisiana, Kentucky, Maine, Nebraska, and Arizona. Most studies came from the Midwest (*n* = 16, 28%), followed by the Northeast (*n* = 6, 11%) and the South (*n* = 5, 9%). Only one study came from the West (Arizona). Figure 4 illustrates the number of studies by area of the world.

We further categorized studies according to the farmworker-related populations specified. Seventeen studies (30%) identified participants as hired workers, while thirteen studies (23%) included more than one population category (e.g., hired workers *and* managers). Four studies (7%) focused on individuals in the farming community, such as family members, and did not explicitly mention their employment status. One study (2%) concentrated on farm operators. Notably, twenty-two studies (39%) did not specify the farmworker-related population.

### Outcomes

**Figure 5:**
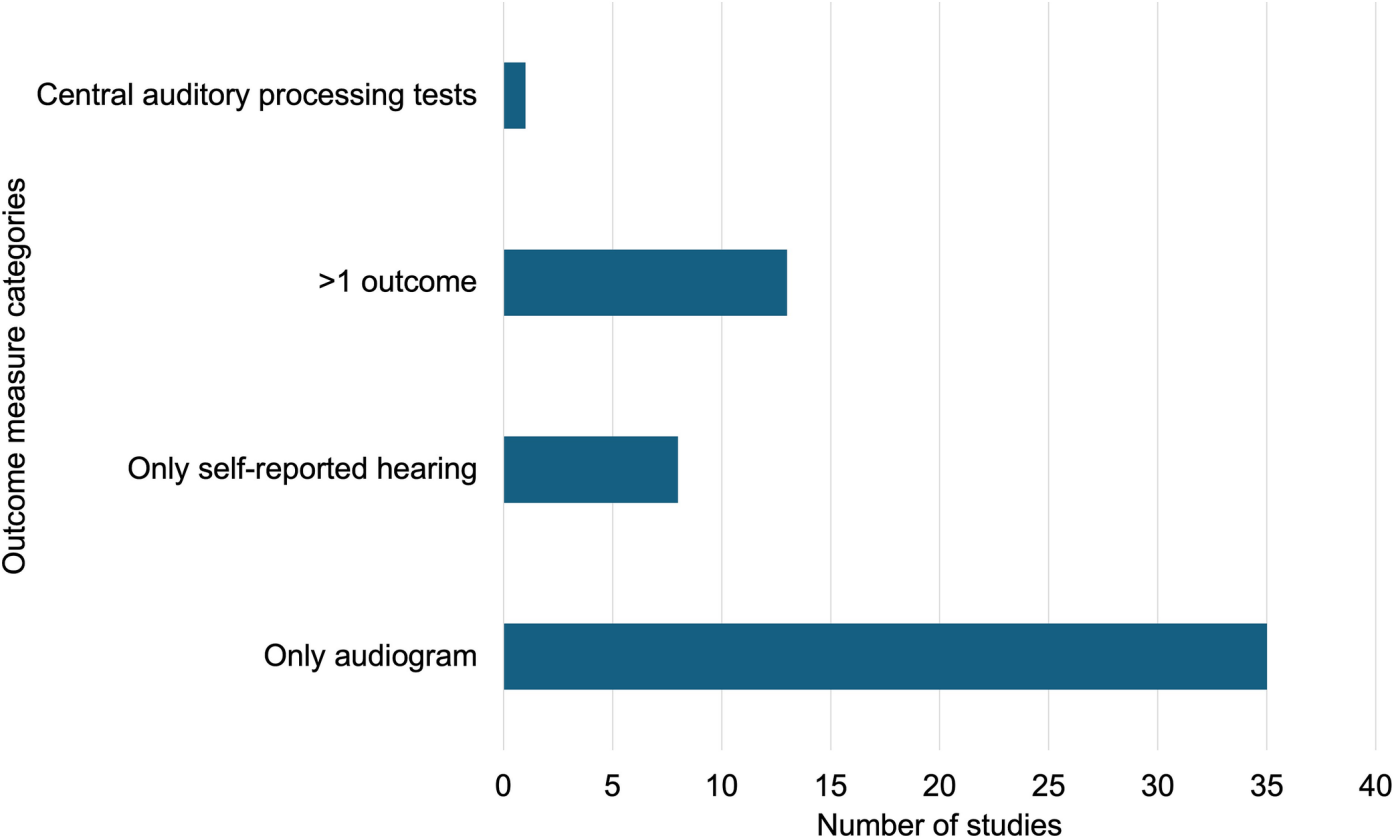
Number of publications by outcome measure category.

All the included articles used quantitative outcomes. More studies focused solely on objective outcomes (*n* = 41, 72%) than on subjective outcomes (*n* = 7, 12%). An additional eight studies included both subjective and objective outcomes. In one additional study, ICD-10 diagnosis codes were used to indicate hearing loss in farmworkers, but it was unclear whether the data were based on subjective or objective information (53). Figure 5 shows the number of studies per outcome measure category.

### Objective outcomes - measures

Among the 49 studies that used objective outcome measures, most (*n* = 47, 82%) used audiometry to evaluate farmworkers’ hearing sensitivity. Hearing loss was primarily described using the pure tone average (PTA). The frequencies selected varied across studies; however, most commonly, studies included a high-frequency pure-tone average of 3, 4, and 6 kHz. The cutoff point for normal hearing also varied across studies. The lowest cutoff was 10 dB HL (54), and the highest was 40 dB HL (55). The cutoff point was typically lower (i.e., stricter) in studies that focused on adolescents. Four studies used audiometry to characterize farmworkers’ hearing sensitivity but did not calculate PTA. Two of these were case studies that described farmworkers’ audiograms in detail as hearing outcomes (47, 48). One study was a large-scale longitudinal analysis of baseline audiogram data compared with audiogram data following a hearing protection intervention (56). One study calculated threshold differences between farmworkers’ ears to evaluate possible asymmetry (44). Two of the 49 studies that included objective measures did not include audiometry: one measured central auditory processing using pitch pattern and duration pattern tests (57), and the other used the digits-in-noise test to measure speech reception thresholds (58).

### Objective outcomes - results

**Figure 6:**
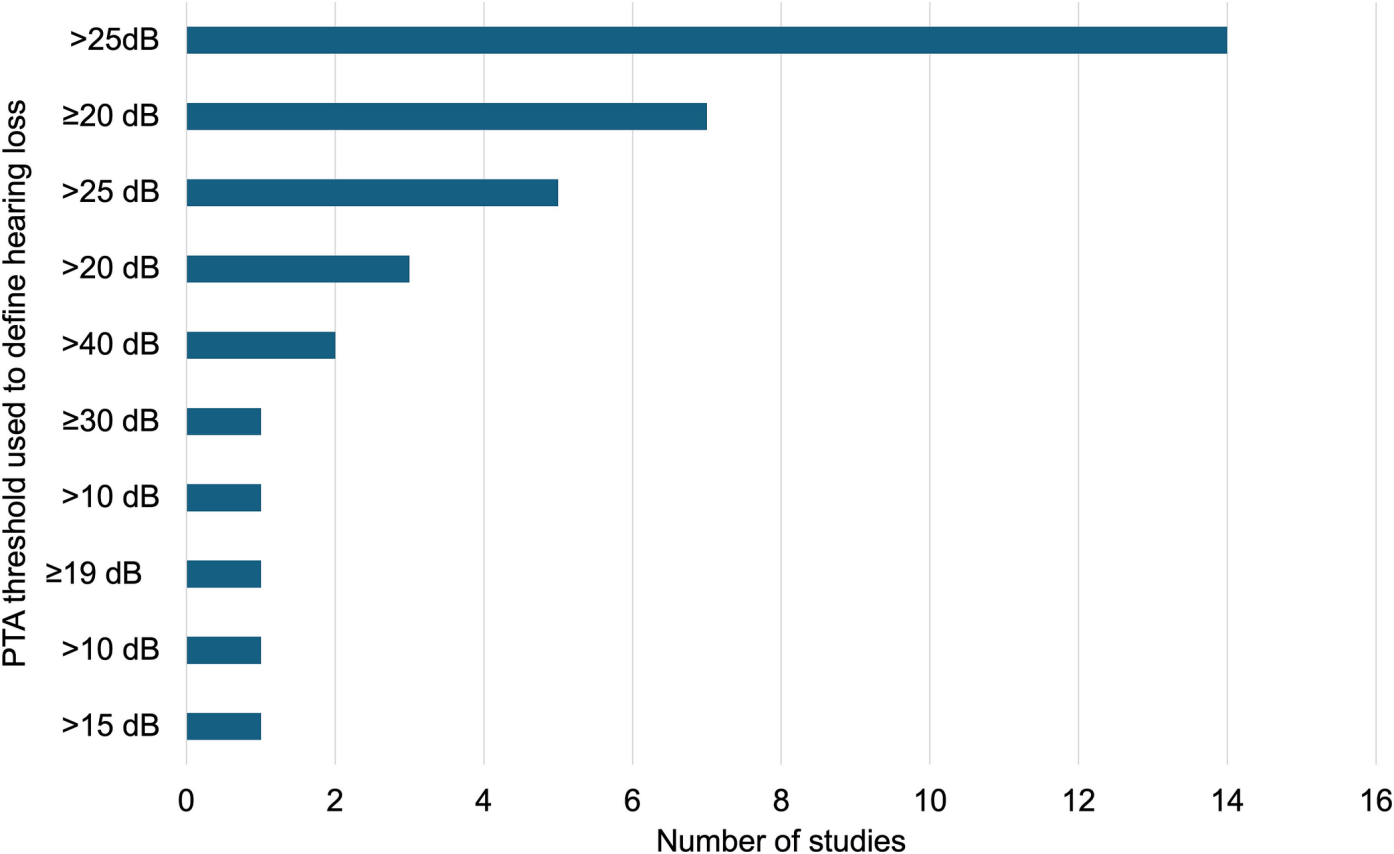
Distribution of publications by Pure Tone Average (PTA) thresholds (dB) used to define hearing loss.

The prevalence of hearing loss among farmworkers in the included studies that used objective measures ranged from 10 to 98%. Figure 6 shows the number of studies by PTA threshold, in decibels, used to determine hearing loss. The most common PTA threshold was greater than 25 dB HL. In the 14 studies that categorized hearing loss as thresholds more than 25 dB HL, the prevalence of hearing loss in farmworkers ranged from 47 to 98%. In seven studies, hearing loss was defined as thresholds greater than or equal to 20 dB HL; in those studies, the prevalence ranged from 38 to 80%. Twelve studies noted that farmworkers’ left ear thresholds were poorer than right ear thresholds. The most common variables associated with more severe hearing loss were age, male sex, and greater number of years working in agriculture. Few studies controlled for age, a common cause of hearing loss. Two studies controlled for age and found that farmworkers were at higher risk for hearing loss even after accounting for this factor (49, 59). Four studies indicated that the use of pesticides increased farmworkers’ risk of hearing loss (2, 8, 48, 60).

### Subjective outcome measures

Subjective hearing problems were primarily measured via researcher-developed questions, although a few studies used published surveys or portions of published surveys. For example, Hwang et al. (61) adapted questions from the 1997 National Health Interview Survey, McCullagh (62) used a 10-item self-administered questionnaire from the National Institute of Deafness and Communication Disorders (NIDCD), and Carruth et al. (63) used items from the Abbreviated Profile for Hearing Aid Benefit [APHAB, Cox and Alexander, (64)].

### Subjective outcomes—results

The lowest prevalence of subjective hearing difficulties was found in Brackbill et al. (65). In that study, farmworkers were asked if they considered themselves “deaf in both ears or other hearing impairments.” Results indicated that the prevalence of reported hearing difficulties was low (0.02%). However, Brackbill et al. noted that age-matched non-farmworkers had an even lower prevalence of hearing difficulties when compared to farmworker participants. The highest prevalence of subjective hearing difficulties across the included studies was found in Johnson (66). In that study, among 84 potato growers in Maine, 87% self-reported hearing loss from loud noise.

### Comparison groups

Nearly half of the studies (*n* = 23, 40%) included a comparison group, typically non-farmers and office workers. Among the studies with a comparison group, all identified a higher prevalence, more significant risk, or greater severity of hearing problems in farmworkers compared to the non-farmworker population. Studies compared farmworkers’ hearing thresholds against thresholds of non-farmworkers in population-based cohorts or enrolled a control group of non-farmworker participants. For example, in Dewangan et al. (67), audiograms of 30 male tractor drivers (mean age = 33.2) were compared against age-matched non-tractor drivers. Results indicated that tractor drivers in the study were nine times more likely to have hearing loss than the control group. In Rabinowitz et al. (18), the prevalence of hearing loss at 4 kHz among male Hispanic/Latino farmworkers was higher than that of a general population of Hispanic/Latino male adults enrolled in the National Hispanic Health and Nutrition Examination Survey (HANES), and this difference was found to exist across every age category. Given that age strongly predicts hearing loss, age-matched case control studies are highly desirable.

### Approaches in data collection

A total of 21 studies (37%) leveraged data from large-scale population-based cohort studies as the primary study population or as the control group (or both). Fourteen cohorts were identified, including the National Health Interview Study, the Iowa Farm Family Health and Hazard Survey, the Agricultural Health Study, and the UK Biobank. Hearing tests in the reviewed studies were conducted in various settings to ensure low background noise and increase the accuracy of the results. Methods included using sound-treated booths mounted in mobile units such as vans and trucks provided by organizations like the Wisconsin Department of Public Instruction and the Red Cross of Japan. Testing locations ranged from farmers’ homes and hospital rooms to quiet rooms within agricultural enterprises and local municipal offices or at field day events. Certified audiologists and technicians used calibrated audiometers in stationary and mobile facilities, including portable hearing booths and custom-made mobile testing units. For example, Karlovich et al. (59) describe testing being carried out in an “otomobile,” a mobile unit provided by the state health department. This otomobile allowed the research team to conduct audiometry testing on-site at farming events while controlling the noise in the environment.

### Common limitations of included studies

Common limitations across the included studies involved either participants or testing. Regarding participants, several authors used a non-random sampling of participants, which could lead to self-selection bias. The studies with smaller sample sizes noted that their results may not be generalizable to the broader farmworker population. Others noted that farmworker participants were healthier and of higher socioeconomic status compared to the entire population of farmworkers. Regarding testing, as mentioned, several studies conducted audiometric testing outside of a typical clinical setting to reach farmworkers at work or at home. Some of these studies indicated that having limited control over their setting may have impacted hearing threshold measurements. In addition, the authors of the included studies mentioned that they could not control for all confounding variables in their analyses, particularly a detailed noise exposure history and accounting for presbycusis.

## Discussion

To our knowledge, this is the first scoping review to describe the extent of NIHL among farmworkers globally. Our review of 57 studies indicated that hearing loss is highly prevalent among farmworkers across various ages and different areas of agriculture. Further, results indicated that hearing loss is more common in farmworkers relative to age- and sex-matched non-farmworkers and increases in severity with more years worked in agriculture. The majority of the reviewed studies were conducted in North America. Approaches to collecting hearing-related data in this traditionally “hard to reach” population included large-scale public health surveillance surveys and meeting farmworkers at their workplace and in their homes.

This review of studies conducted over the past 66 years highlights that the problem of hearing loss in this population has not decreased over time. Our current review updates the findings of McCullagh (33), which summarized the literature on hearing loss among US-based agricultural workers up to 2001. Notably, our study included a global perspective, which allows us to compare testing approaches and the prevalence of hearing loss among a diverse group of farmworkers. Since 2001, there has been a notable increase in the number of publications on farmworkers’ hearing loss, including the addition of population-based and longitudinal studies. These study designs help improve our estimates of NIHL in farmworkers because they help differentiate the impact of noise on hearing loss from other variables, such as age and noise exposure from other sources, including jobs or hobbies. However, comparing the two reviews reveals a lack of progress in other areas. In 2002, McCullagh (33) emphasized that there was a need for studies focused on agriculture’s highest-risk groups, including women, seasonal workers, and children, as well as participant populations that reflect the high proportion of minority farmworkers in the US. McCullagh (33) also highlighted the importance of documenting noise exposures according to the type of agricultural operation, geographic region, and crop type to understand better how hearing loss affects different populations. Unfortunately, it appears that these research gaps still exist more than two decades later.

Characteristics of the 57 studies included in our scoping review are discussed below in detail, including study design, outcomes, participants, approaches in data collection, and common limitations. In each area, we provide specific recommendations for future research based on the results of our review.

### Study design

Although the majority of studies used a cross-sectional design, eight studies used a longitudinal design, in which farmworkers’ hearing sensitivity was measured across more than one time point. This study design is generally considered of higher scientific quality and can improve our precision of NIHL measurements, but there are also limitations. Longitudinal study designs are challenging to carry out among farmworkers who may change location often. While perhaps challenging to reach, this population makes up most of the agricultural workforce in many regions, making it imperative not to exclude them from research efforts.

Several studies utilized large-scale public health surveillance studies, which offer the advantage of large sample sizes but also have limitations. For instance, participants in the UK Biobank sample are more likely to be of higher socioeconomic status and have higher levels of education compared to the general population (58). We also found a lack of well-controlled studies that account for confounders influencing hearing, such as age. This will be examined in more detail in a future meta-analysis.

We recommend adopting research approaches that balance the rigorous scientific standards of longitudinal designs and the inclusivity necessary to represent diverse populations accurately. Researchers can adapt longitudinal study designs to accommodate the transient nature of the farmworker, such as by leveraging mobile health technologies and establish partnerships with trusted community-based organizations to facilitate participant engagement and retention across multiple locations. Further, the increase and dependance on H-2A visa program workers could offer a way to measure hearing loss in this population systematically.

### Outcomes

Among studies in this review, hearing loss was prevalent among farmworkers as measured by audiometry. However, the frequencies tested and the threshold for what was considered normal vs. abnormal hearing varied, making it difficult to compare outcomes across studies. To facilitate comparison, we recommend that future studies using audiometry evaluate farmworkers’ hearing thresholds at the following frequencies, at a minimum: 0.5, 1, 2, and 4, and 6 kHz. This allows researchers to calculate a four-frequency PTA at 0.5, 1, 2, and 4 kHz, as recommended by the World Health Organization, as well as account for hearing changes across broader range of frequencies affected by noise, as recommended by agencies such as the National Institute for Occupational Safety and Health (68). Hearing loss should be defined as specified by the World Health Organization: 20–34 dB mild, 35–49 dB moderate, 50–64 dB moderately severe, 65 to 70 dB severe, and 80–94 dB profound (69). Studies may also consider including additional PTA calculations and hearing loss cutoff points to meet their research’s specific needs.

Regarding subjective outcomes, researchers are encouraged to publish the measures or questions used in their study, along with any psychometric testing conducted on those measures, to improve the rigor, reproducibility, and generalizability of findings. There was a notable lack of qualitative outcomes in the studies reviewed. Qualitative outcomes can help understand the thoughts and experiences of farmworkers’ hearing loss, and the social determinants of health, which is particularly important for vulnerable farmworker populations. We recommend that future studies incorporate mixed methods research that combines quantitative data with qualitative insights gathered through interviews or focus groups. This approach can help us understand how noise and hearing loss impacts farmworkers’ quality of life. Additionally, we recommend collecting information on farmworkers’ areas of agriculture, primary crops, roles, and equipment used, as well as health-seeking behaviors and information about access to health care. Such information can help improve our understanding of hearing loss, which is more severe in certain areas.

### Participants

Although various geographic regions were represented in this review, there was a notable lack of research in areas with high agricultural activity, including China, Indonesia and Ethiopia. From the US, studies from California and Texas were absent, two states with large populations of migrant Hispanic/Latino farmworkers (70). Additionally, most studies did not report participants’ race or ethnicity, making it difficult to determine if the results are generalizable or representative of the wider farmworker population. Furthermore, research among female farmworkers and migrant/seasonal farmworkers was lacking despite these groups potentially having unique risks and needs related to NIHL.

### Approaches in data collection

Researchers conducted testing at worksites, homes, and various other non-clinical locations, reflecting the unique challenges of reaching farmworker populations. To balance the need to meet people where they are while maintaining rigorous testing standards, we recommend using mobile testing units outfitted, when possible, with sound attenuation or sound-treated booths. This approach has proven effective in previous studies included in this review and should be expanded to cover more regions and populations. Additional strategies for connecting with hard-to-reach farmworker populations include partnering with Community Health Workers, offering bilingual services, and teaming up with other social services in the community. Additionally, future research should consider incorporating new technologies, such as telehealth-enabled systems, to facilitate remote data collection and engage a diverse range of participants (71). Results revealed that most included studies lacked data on the personnel conducting audiometry testing. Therefore, we recommend that future studies provide detailed information on any training, experience, and qualifications of the testing personnel, such as whether they are certified audiologists, trained technicians, or community members. Such information is crucial for evaluating the reliability and consistency of the data and will assist future studies in planning and allocating resources.

### Strengths, limitations, and future directions

A major strength of this review was in the comprehensive approach to understanding hearing loss in farmworkers globally. Our review synthesized data across a wide range of variables, including population demographics, hearing loss outcomes, and data collection methodologies. Another strength lies in our use of rigorous and transparent methods, including Arksey and O’Malley’s framework, the PRISMA-ScR guidelines, and valuable guidance from an experienced health sciences research librarian.

Our findings are also subject to the following limitations. First, migrant and seasonal farmworkers are underrepresented, and therefore our results cannot be generalized to the entire farmworker population. Next, many studies did not specify whether the farmworker population included hired workers, owners/operators, or unpaid family members. These populations differ significantly in terms of social determinants of health, including housing stability, economic access, occupational hazards, and access to healthcare, which could influence the prevalence and severity of hearing loss. Additionally, our review only included articles published in English, meaning we may have missed relevant studies available exclusively in non-English languages. Lastly, the nature of scoping reviews, which do not include a quality assessment phase, means that some of the included studies may have been of poor or moderate methodological rigor. Future research, particularly a meta-analysis, is warranted to clarify trends in hearing loss prevalence over time, focusing on studies with high methodological rigor.

## Conclusion

Agriculture is an exceptionally high-risk industry, where farmworkers face significant safety and health hazards due to heat, dust, chemical exposure, and machinery operations, all of which contribute to illness and injury. Among these hazards, hearing loss from exposure to loud machinery or equipment is a common yet often overlooked issue. Hearing assessments are crucial for identifying the health impacts of noise and other environmental exposures, the effectiveness of intervention efforts, and supporting public health surveillance efforts. The evidence from this review indicates that hearing loss is prevalent among farmworkers, including those involved in crop and animal farming, as well as tractor drivers. Our review also found a significant variation in how researchers quantify hearing loss and a lack of reporting details on population demographics, which limits our ability to understand the generalizability of results and complicates efforts to compare different groups. Looking ahead, better coordination of research methodologies across studies could facilitate a more accurate and comprehensive understanding of hearing loss distribution among farmworkers.

Researchers should aim to design studies around standardized outcomes, although we acknowledge that this is challenging due to the lack of consensus on the definitions and metrics used to describe NIHL. Furthermore, our review highlighted that research among vulnerable populations, including Hispanic/Latino migrant and seasonal farmworkers who make up a substantial portion of the US agricultural workforce, is very limited. Environmental health research increasingly emphasizes the importance of partnering with communities to engage vulnerable populations in research and address health inequities. This approach can help build trust and enhance research quality by ensuring that research is grounded in the experience of those affected by the health issue. Researchers should focus on using a community-engaged approach to enhance research among under-represented farmworker populations to ensure that data are inclusive and representative, which in turn help to protect the hearing health of all farmworkers better and guide more informed policy and practice decisions in occupational health.
