# Supplementary material for: Noise-induced hearing loss in farmworkers: a scoping review
Source: Front Public Health. 2025 Mar 3;13:1502489. doi: 10.3389/fpubh.2025.1502489 (PMC11913118; doi:10.3389/fpubh.2025.1502489)
Supplement: Supplementary file 2 [file Data_Sheet_2.PDF]

Appendix Table 2. Use of hearing protection and summary of main findings among studies that did not report source of noise exposure (n = 25)

Last name, first author	Title	Use of hearing protection (type, frequency)	Main Findings
Thelin	High-frequency hearing loss in male farmers of Missouri	not reported	Hearing loss at 2 and 4kHz was higher for farmers than for office workers (16.8% vs 6.2%).
Karlovich	Hearing sensitivity in farmers	18-25% males, 9-15% females reported using hearing protection	Hearing was poorer with increasing age and at higher frequencies; hearing loss between 3-6kHz was worse for males vs females at all ages. High-frequency hearing loss was similar for farmers and non-farmers and could not be attributed to age alone. An estimated 25% of males have hearing loss by age 30, 50% by age 50.
Plakke	Occupational hearing loss in farmers.	not reported	Farmers exposed to noise have poorer hearing than individuals not exposed to noise. Among 25–34-year-olds, hearing loss was 10%, among 35–44-year-olds, 30%, and among 45-54 year olds, 50%. "
Brackbill	Prevalence of chronic diseases and impairments among US farmers, 1986-1990.	not reported	The prevalence of hearing loss was higher for farmworkers (vs other workers) only among the oldest age group (older than 65 years old).
Eddington	Noise induced hearing loss amongst Australian farmers	not reported	Farmworkers ages 35 and greater had more hearing loss than office workers. Greater hearing loss was associated with firearm use and holding multiple jobs among farmers.
Miyakita	Estimates of workers with noise-induced hearing loss and population at risk	not reported	The estimated number of agricultural workers who have hearing loss more than 40 dB at 4kHz is 360,000, or 10% of all agricultural workers.
Varchol	Farm residence increases risk of hearing loss among youth	not reported	At 6kHz, the hearing thresholds of the farmworkers exceeded the control group by almost 6dB. High-frequency PTAs for farm youth were higher than non-farmworkers, indicating farmworkers are at a higher risk for hearing loss (RR: 1.87)
Lupescu	Hearing conservation program for farm families: an evaluation	Males 47% n=507; Females 18% n=58 (of both males and females who reported any hearing protection use >60% used hearing protection less than half the time they were exposed to noise.	31% of farmworkers had "early" hearing loss, defined as a notch of at least 15dB at 3, 4, or 6kHz.

Firth	Health of farmers in Southland: An overview.	not reported	28.7% of farmworkers ages 45 and older had hearing loss.
Gomez	A comparison of self-reported hearing loss and audiometry in a cohort of new York farmers	not reported	36% of farmworkers had at least some difficulty hearing in one or both ears, audiometric hearing loss was 9% for binaural low-frequency PTA, 29% for binaural mid-frequency PTA, and 47% for binaural high-frequency. Self-report and audiometry had the best agreement at binaural mid-frequency PTA.
Kerr	Perceived and measured hearing ability in construction laborers and farmers	not reported	At 4kHz, 67% of farmworkers had hearing loss and 53% of construction workers had hearing loss. Perceived hearing loss was worse among those with measured hearing loss, although there was a poor relationship between perceived and measured hearing loss. Hearing loss was worse in males.
Choi	A Comparison of Self-Reported Hearing and Pure Tone Threshold Average in the Iowa Farm Family Health and Hazard Survey	not reported	Prevalence of bilateral hearing loss according to the audiogram was 49.5%, higher in the oldest age group. Among older farmworkers, the prevalence of hearing difficulties using the self-report measure was lower than audiogram.
Crawford	Hearing loss among licensed pesticide applicators in the agricultural health study.	not reported	Self-reported hearing loss was 35%, prevalence increased with age. Applicators from Iowa had a greater odds of hearing loss vs. those from North Carolina. Exposure to pesticides was associated with increased risk of hearing loss.
Hoshino	Ototoxicity study in workers exposed to organophosphate	22.2% used PPE (masks, gloves, and "proper apparel")	16 workers had irritative peripheral body balance disorder and 7 had sensorineural hearing loss
Berg	Hearing conservation program for agricultural students: Short-term outcomes from a cluster-randomized trial with planned long-term follow-up	classroom instruction, distribution of hearing protection devices, direct mailings, noise level assessments, and yearly audiometric testing	All farm youth participants have hearing loss at 6kHz but there does not appear to be any reduced level of NIHL over the 3-year follow-up period among farm youth randomized to the hearing conservation program.
Johnson	Study finds self-report valid for assessing hearing loss incidence in farm population.	not reported	85.6% of farmworkers had audiometric hearing loss, and 87% of farmworkers self-reported hearing difficulties. There was good alignment between self-report and audiometric data.
Guida	Audiological Evaluation in workers exposed to noise and pesticide	ear plugs	Farmworkers exposed to both pesticides and noise had worse hearing thresholds according to audiometry when compared to farmworkers exposed to only noise.

McCullagh	Validation of a questionnaire to identify hearing loss among farm operators	not reported	Overall, the NIDCD questionnaire did not have good agreement with audiogram results. The single item from the questionnaire "Do you have to strain to understand conversations" had moderate agreement with screening audiogram outcomes. More than 70% of the sample "failed" the questionnaire (indicating hearing loss) and audiometry revealed mean binaural thresholds at 4kHz at 38dB.
Solecki	Hearing loss among private farmers in the light of current criteria for diminished sense of hearing.	not reported	78% of farmers had hearing loss over 20dB in the high frequencies, versus in the control group, 17% had high-frequency hearing loss. In farmers, hearing loss was associated with age and period of work.
Canton	The consequences of noise-induced hearing loss on dairy farm communities in New Zealand	not reported	Self-reported hearing difficulties were identified in 48% of dairy farmworkers. Hearing loss negatively impacts quality of life among farmworkers.
Masterson	Prevalence of hearing loss among noise-exposed workers within the agriculture, forestry, fishing, and hunting sector, 2003-2012	not reported	The overall prevalence of hearing loss across sectors of agriculture, forestry, fishing, and hunting was 15%. Among crop agriculture workers, the prevalence was 13.18%, among animal agricultural workers, it was 16.55%. Prevalence varied by sub-groups.
Arve	Simplified risk assessment of noise induced hearing loss by means of 2 spreadsheet models	not reported	Higher prevalence of high-frequency hearing loss among farmworkers (18.3%) compared with reference group (teachers, 14.6%).
Lee	Hearing impairment among Korean farmers, based on a 3-year audiometry examination	not reported	The prevalence of moderate to profound hearing loss was 19.6%. Hearing loss increased with age. Hearing loss at 3kHz was more prevalent in men than women.
Coco	Hearing Difficulties Among Farmworkers in the México-US Southwest Border Region.	not reported	36% of participants reported they have or might have hearing difficulties.
van der Molen	Trends in occupational diseases in the Italian agricultural sector, 2004–2017	not reported	The incidence of hearing loss in the agricultural sector increased 7% between 2014 and 2017.

Appendix Table 3. Use of hearing protection and overview of main findings among studies that reported sources of noise exposure (n = 32)

Last name, first author	Title	Source of noise and dB level if available	Use of hearing protection (type, frequency)	Main Findings
Lierle	The effect of tractor noise on the auditory sensitivity of tractor operators	Tractors (working & idling; 88-102.5 SPL in the 300-600 cycle octave band and 85-98 SLP over the 600-1200 cycle octave band)	not reported	Tractor drivers had worse hearing at 1kHz vs general population, tractor operators had greater "dips" in hearing at 4kHz, particularly among 30- 60 year olds.
Lawhorne	The health of farmers	Tractors	not reported	Younger farmers had hearing loss at >25 dB, older farmers had hearing loss even when controlling for presbycusis; hearing loss increased with tractor time.
Pfeiffer	Aspects of physical fitness and health in Ontario dairy farmers	Engine and motor noise from heavy agricultural machinery	not reported	Increase in thresholds of 4 dB between each age decade, increase in thresholds at 4 and 6kHz.
Kristensen	Occupational hearing impairment in pig breeders.	Pigs at 94-104 dBA, system for grinding and mixing pig fodder at 88 dBA, high-pressure cleaner 98-105 dBA	none used	Farmer had severely sloping normal to profound bilateral sensorineural hearing loss that was though tot be the result of noise related to pig breeding.
Broste	Hearing loss among high school farm students.	Tractor, Combine, Compressor, Elevator, Haybine, Chopper, Silo Unloader, Ginder, Chain Saw, Motorcycle/Snowmobile, Pistol/Rifle, Woodworking Equipment, Amplified Music	9-11%	Farm youth with more experience farming have a higher proportion of hearing loss. 71% of farm youth who live on farms and participate in farming activities had hearing loss.
May	Noise-Induced Hearing Loss in Randomly Selected New York Dairy Farmers	Tractors and other farm implements	<20%	37% of participants had PTAs $\geq$20dB in either ear, and 65% had high-frequency PTAs $\geq$20dB. The left ear was worse than right. Hearing loss was related to years worked and age.
Marvel	Occupational hearing loss in New York dairy farmers.	Farm machinery (tractors full throttle no cab 83-104 dBA, tractors full throttle with cab 77-88 dBA, feed carts 85-93 dBA, bedding choppers 94-102 dBA, milkhouse 72-94 dBA, chainsaws 105-115 dBA) also snowmobiles and ATVs for both work and recreation	participants "tend not to wear hearing protection" (p. 525)	A higher proportion of farmers had hearing loss in high and mid-frequencies versus non-farmers (high: 65% vs 37%; mid: 37% vs. 12%). Hearing loss was related to years worked and age. Left ears worse than right.

Solecki	Occupational hearing loss among selected farm tractor operators employed on large multiproduction farms in Poland	tractors and self-propelled agricultural machines	not reported	Farm tractor drivers had worse hearing from 3-6kHz versus control group. Hearing loss at > 20dB was found among 56% of farmworkers at high-frequency PTA, and 22% of farmworkers at mid-frequency PTA, no hearing loss in the control group. Hearing loss strongly correlated with number of years working.
Beckett	Hearing conservation for farmers: Source apportionment of occupational and environmental factors contributing to hearing loss.	Firearms, tractors, combine, chain saws, grain dryers. Tractors (90.7 avg dBA), Milk Area (76.4 avg dBA), Milk House (82.2 avg dBA), Vacuum Pump (91.9 avg dBA), Milk Cooling Compressor (83.8 avg dBA), Bedding Chopper (93 avg dBA), Fruit/Vegetable Processing Area (83 avg dBA), Fruit Vegetable Utility Room (83.5 avg dBA), Feed Unloading Area (90.4 avg dBA).	not reported	Nearly all of the farmworkers (98%) had hearing loss. Hearing loss was associated with age, male sex, education, years hunting with guns, years using a grain dryer, and history spraying crops with pesticides in the last year.
Hwang	Predictors of hearing loss in New York farmers	Farm noise above 85 dBA (tractors, combines, chainsaws, grain dryers), Firearm use (39%, not found to significantly correlate with hearing loss)	not reported	22% of farmworkers reported at least some trouble hearing in one or both ears. Age, sex, being from a livestock farm, loss of consciousness due to head trauma were all associated with hearing loss.
Teixeira	Occupational Exposure to insecticides and their effects on the auditory system	motorized pumps (>90 dB)	34.7% used PPE (included respirators, glasses, hearing protection, helmets)	A greater proportion of farmworkers who were exposed to insecticides had central auditory dysfunction (56%) compared to farmworkers who were unexposed (9.8%, RR = 7.8)
Stewart	Perceived effects of high frequency hearing loss in a farming population.	Farm noise (100% of participants), firearms (72%), other occupational (e.g. factory) work (32.2%), noisy hobbies (e.g. snowmobiles, fireworks) (35.4%), loud music (21.5%)	60% participants reported using hearing protection for an average of 34.5% of the time they were exposed to loud farm noise	Farmworkers over 50 years of age had significantly more hearing loss vs. younger farmworkers; part-time workers had poorer high-frequency hearing loss vs. full-time farmworkers, potentially because of the equipment they work with. Hearing loss worse at 6kHz.
Miyakita	Noise exposure and hearing conservation for farmers of rural Japanese communities	Harvesting and processing machines (exceeding 8hrs/day of exposure to sound levels >85 dBA in 19/23 cases)	not reported	A greater proportion of farmworkers have hearing loss at 40dB at 4kHz versus office workers. Among participants in their 40s, 16.4% of farmworkers had hearing loss vs 9% of office workers. For participants in their 60s,

				50.3% of farmworkers had hearing loss vs 29.9% of office workers.
Rabinowitz	Hearing loss in migrant agricultural workers	Machinery (35.3% of participants), tractors (36%), firearm use (21.3%), noisy job rest of the year (35.3%), loud music (58%)	14% participants reported use of hearing protection	More than half of participants had hearing loss between .5-6kHz, association between Hispanic ethnicity and reported hearing difficulty; farmworkers had greater prevalence of hearing loss vs. control group (HHANES); abnormal middle ear dysfunction in 19% of sample; this dysfunction associated with greater risk of hearing loss and subjective hearing difficulty.
Kumar	Effect of tractor driving on hearing loss in farmers in India	Tractors (>100 dBA), electric pump sets (>90 dBA), diesel pump sets (>90 dBA), fodder cutter machines (>90 dBA), flourmills (>90 dBA), thresher (>90 dBA), sugar cane crusher (>90 dBA) for over 8hr/day	none used	Four (of 50) farmworkers in each group self-reported hearing difficulties. Audiograms showed more severe hearing loss in tractor drivers versus non-tractor driving farmworkers who were exposed to less noise.
Carruth	The impact of hearing impairment, perceptions and attitudes about hearing loss, and noise exposure risk patterns on hearing handicap among farm family members	Lawn mowers/weed trimmers (44.6% of participants), power tools (41.1%), tractor without cab (39.3%), four-wheelers (35.7), hunting or shooting guns (17.9%), tractor with cab (16.1%), loud music (16.1%), dairy pumps (12.5%), hog confinement building (10.7%), boats (7.1%), Military service (7.1%), playing in a band (1.8%)	7.1% (n=4/56) wore hearing protection >50% of the time at work, 7.1% (n=4/56) reported wearing hearing protection during recreational noise exposure. Ear plugs were the only type of protection reported.	80.4% of farmworkers and their family members had high-frequency hearing loss. Left ear hearing loss poorer than right.
Gates	A Pilot Study to Prevent Hearing Loss in Farmers	Impact or impulsive noise exposure levels at or above 140 dBA (including tractors, air grinders, fans, and other equipment)	At Baseline: 60% (n=15) participants reported never using hearing protection while farming, 28% (n=7) reported seldom using hearing protection, 4% (n=1) reported sometimes using hearing protection, 4% (n=1) reported often using hearing protection, 4% (n=1) reported always using hearing protection.	36% of farmworkers currently have or have had a hearing problem related to farming.

Renick	Hearing Loss Among Ohio Farm Youth: A Comparison to a National Sample	Loud music (24.2%), augers (14.4%), tractors without cabs (12.1%), and lawn mowers (9.8%)	not reported	Farm youth had a higher prevalence of hearing loss when compared to a national comparison sample, particularly at 6kHz (~50% of farm youth had hearing loss).
Depczynski	Changes in the hearing status and noise injury prevention practices of Australian farmers from 1994 to 2008.	Firearms, chainsaws, workshop tools, heavy machinery, tractors with cabins, uncabbed tractors	Improvement in the frequency of hearing protection use between two time periods for specific high-risk activities such as chainsaw use and firearms.	Improvements in noise protection practices over the 14-year period in the study was evident in the reduction of mean hearing thresholds for all ages groups in sample
Humann	Hearing loss and task-based noise exposures among agricultural populations	Hunting/target shooting, ATV or motorcycle, chain saw, pneumatic or electric tools, tractor without a cab, tractor/combine with a cab, grain dryer, feed mill, hay chopper, livestock, hog confinement building	Never 19 participants (59.4%), Some of the Time 9 (28.1%), Most of the Time 3 (9.4%), All of the Time 1 (3.1%)	Farmers have greater hearing loss than non-farmers, self-reported years of work in specific tasks was poorly associated with hearing loss.
Depczynski	Meeting national targets for preventing noise injury in young farmers (15-24 years)	Tractors, chainsaws, firearms, workshop tools	At baseline: while driving a tractor without cab 25.7% of participants always use protection, 34.7% sometimes, 39.7% never. While operating a chainsaw, 34.9% participants always use protection, 25.6% sometimes, 39.5% never. While using firearms, 11.3% of participants always use protection, 24.0% sometimes, 64.7% never. While using workshop tools, 19.3% always, 31% sometimes, 49.7% never. In other situations, 9.1% always, 29.4% sometimes, 61.5% never.	In male farmworkers, the proportion of farmworkers with normal vs noise-induced hearing loss improved significantly in both right (from 61.8% to 80.5%) and left ears (from 62.0% to 76.3%); in female farmworkers, the proportion of farmworkers with normal vs noise-induced hearing loss also improved significantly in both right (from 68.4% to 77.2%) and left ears (from 57.1% to 60.4%).
Humann	Effects of common agricultural tasks on measures of hearing loss	Hunting/target shooting, all-terrain vehicle (ATV)/motorcycle riding,	not reported	Specific agricultural tasks significantly associated with HL including hunting or target shooting, ATV or motorcycle riding, chain saw

		chain saw, electric/pneumatic tool use, farm noise, and agricultural tasks		use, living on farm, and other agricultural tasks - but only for males. Average PTA for males PTA = 32.6 dB HL; females PTA = 20.0 dB HL
Berg	Asymmetry in noise-induced hearing loss: evaluation of two competing theories	Occupational-agricultural work, firearms-gunshots	Frequency of use (guns) Never 33.5%, Sometimes 7.8%, Often 13.6%, Frequently 18.9%, Always 26.2% (Agriculture) Never 35.1%, Sometimes 37.2%, Often 15.7%, Frequently 10.5%, Always 1.6%	At frequencies higher than 2kHz, men had more hearing loss and more asymmetry, and a different asymmetry pattern, than women. There was greater susceptibility to the left ear.
Khadatkar	Hearing Impairment of Indian Agricultural Tractor Drivers	Tractors (six different models; one tractor was 4-wheel drive and 5 tractors were 2-wheel drive)	not reported	Audiometric thresholds of tractor drivers were worse than that of a comparison group (office workers). Tractor driving is estimated to add 7.1% excess risk of hearing loss.
Sena	The hearing of rural workers exposed to noise and pesticides	Rudimentary motor vehicle (noise measured in this vehicle was 88.3 dBA up to 93.4 dBA)	not reported	The farmworker who was exposed to pesticides had elevated high-frequency thresholds person had elevated high frequency audiometry thresholds
Ehlers	Hearing Loss and Hearing Protection Use Among Midwestern Farmers	Farm equipment, all terrain vehicles, livestock, firearms, self-propelled equipment	earmuffs, earplugs	At baseline (pre-intervention) the average high-frequency PTA was 33.9dB among control group farmworkers and 23.6dB among intervention farmworkers. After four years, there were seven standard threshold shifts, most of which occurred in the left ear. Farmers' high-frequency PTA did not differ between groups following the educational intervention.
Khadatkar	Effect of age and duration of driving on hearing status of Indian agricultural tractor drivers	Tractor	not reported	Hearing loss exceeded 25dBA for tractor drivers, but not for office workers except at the highest age group. Among tractor drivers, hearing loss was associated with age and driving experience (greater than or equal to 15 years).
Choochouy	Hearing loss in agricultural workers exposed to pesticides and noise	Rice blower, Hand tractor, Riding tractor, Hand grass mower	not reported	Aging, smoking, increased use of pesticides (insecticide or organophosphate), use of agricultural machinery shown to contribute or be predictors to hearing loss.

Couth	Hearing Difficulties and Tinnitus in Construction, Agricultural, Music, and Finance Industries: Contributions of Demographic, Health, and Lifestyle Factors	Recreational noise (music), occupational noise	not reported	The prevalence of tinnitus was 18.59% in agricultural workers. The presence of hearing loss (defined as SNR above -5.5dB according to the digits in noise test) was 14.36%.
Fern	Factors associated with work accidents in a rural area in Minas Gerais Brazil	Tractors, agricultural machinery, hand-held power tools, handling animals, use of agricultural trailers and pesticides	not reported	Noise-induced hearing loss was associated with increased occurrence of work accidents (OR 38.60).
Farfalle	Exposure to Solvents and Noise as a Risk Factor for Hearing Loss in Agricultural Workers	Tractor, combine, implements, power tools, and other noise.	not reported	Exposure to both solvents and noise increased odds of moderate to severe hearing loss (OR 6.03).
Dungan	Noise exposure and hearing loss among tractor drivers in India	Tractors	not reported	The prevalence of high-frequency hearing loss was higher among tractor drivers than control group participants (50% vs 10%).
